# Study on the Influencing Factors of Online Learning and Its Operation Mechanism among Chinese College Students during the COVID-19 Pandemic—An Empirical Analysis Based on a Mixed Method

**DOI:** 10.3390/ijerph192215161

**Published:** 2022-11-17

**Authors:** Huanhuan Li, Bingyu Duan, Yifang Wang, Huijuan Di, Hongxuan Ge

**Affiliations:** 1 College of Educational Science, Xinjiang Normal University, Urumqi 830017, China; 2 Shanghai Institute of Early Childhood Education, Shanghai Normal University, Shanghai 200234, China; 3 Department of Preschool Education, Hebei Normal University, Shijiazhuang 050024, China

**Keywords:** grounded theory, online learning, influencing factors, mixed method, China

## Abstract

Online learning has gradually become a mainstream teaching method to replace traditional classroom teaching during the COVID-19 pandemic. However, there are some issues such as the lack of theoretical guidance on online learning, and the influencing factors of online learning effect are not well proven. On this basis, this study aimed to construct and test a theoretical model of online learning influencing factors among college students through a mixed-method approach in China to further explore the influencing factors of online learning and the relationship between these influencing factors. First, 130 college students were interviewed from May 2021 to November 2021, and the collected data were coded to a theoretical model through grounded theory. Second, we tested and verified the theoretical model through the analysis of survey data of 225 college students from January 2022 to July 2022. Lastly, the results indicate that the learning expectation influences the learning result through learning quality or learning interaction and their combined effects. These findings can help educators around the world gain insight into the process of online learning and improve the quality of online learning during the COVID-19 pandemic.

## 1. Introduction

Statistics show that a total of 1.58 billion students worldwide were not able to return to school due to the COVID-19 pandemic in 2020, and schools in 195 countries were forced to close as a result [1]. Before COVID-19, traditional institutions of higher education had a variety of options for instructional practices, but they also had to shift overnight from a face-to-face model to a fully online model [2]. For example, ministries of education in Latin American countries published notices that suspended traditional classroom instruction in higher-education institutions and implemented online courses during the COVID-19 pandemic [3]. To ensure the availability of online teaching and learning in different quadrants in Indonesia, SPADA Indonesia (Indonesian Online Learning System) was developed collaboratively by the Ministry of Research, Technology, and Higher Education, Directorate General of Learning and Student Affairs [4]. The United Nations Educational, Scientific, and Cultural Organization (UNESCO) also stated that online learning will become the “new normal” for future education [5]. 

The concept of online learning was first introduced by Hiltz [6]. He defined online learning as the construction of a virtual network learning space by putting course materials on a shared network. With the development of technology and the outbreak of the COVID-19 pandemic, there are new changes in the form and definitions of online learning. For example, Singh and Thurman [7] defined online learning as “learning experiences in synchronous or asynchronous environments using various devices with Internet access”. According to the Chinese researcher Gui [8], online learning refers to a remote teaching and learning method based on Internet technology, which can break the boundaries of time and space, as well as present and deliver learning content through the Internet and computers, to realize the remote communication between teachers and students in both directions. To sum up, we can consider the basic connotation of online learning as a way of learning in which students discuss course content with teachers and peers through computer equipment and an Internet-based virtual environment.

Online learning has advantages, including breaking the time and space constraints, flexibility in the learning methods, the support of both synchronous and asynchronous lectures, rewatchable learning content, and retraceable learning processes, which are of key importance for university education during the COVID-19 pandemic [9]. In recent years, researchers have also attempted to explore the influencing factors of students’ online learning such as assessing the relationship between student learning performance and social media usage through the constructivist theory during the COVID-19 pandemic. The findings indicated that online learning mediated the relationship between student interactivity and satisfaction during the COVID-19 pandemic, and that collaborative learning and student engagement using social media had a direct positive impact on student–peer and instructor interactions. Furthermore, student interactions with peers and teachers had a direct positive impact on online learning [10]. A study [11] discovered that individual characteristics specific to each student, such as the student’s knowledge, needs, and preferences for the quality of online education influence the students’ behavior and attitude. Despite the rapid development of online learning promoted by the COVID-19 pandemic, there were still many problems in the online learning process. For example, relevant research has found that, when students face boring online lectures, insufficient auxiliary equipment, and poor network quality, it decreases their learning participation [12]. In addition, some scholars have conducted research on the role of factors influencing online learning. For example, Sarfraz et al. [13] conducted a study on medical students’ online learning, which found direct and indirect impacts of the learning perceptions on learning outcomes through their preparation for online learning. They also identified the moderating role of teachers’ online teaching preparation on medical students’ online learning perceptions and learning outcomes. Miao and Ma [14] explored the correlation among online interaction, self-regulated learning, social presence, and online learning engagement. The results of the study suggested that online interaction and self-regulation influence social presence, and that social presence mediates online interaction and learning engagement, as well as mediates self-regulation and learning engagement. In addition, some factors, such as social isolation and economic recession, bring anxiety and stress to students [15], which in turn affects their online learning effectiveness [16].

According to the relevant research, the exploration of the influencing factors of students’ online learning is relatively scattered, the related research methods are relatively simple, and the discussion on the mechanism and the relationship between the influencing factors is relatively simple and lacking. Therefore, to fill this gap, this study adopts a combination of qualitative (grounded theory) and quantitative (structural equation model technique) research to construct and test a theoretical model of online learning among college students. Two research questions are addressed:

1. What are the factors that influence college students’ online learning?
2. What is the relationship between these influencing factors?

We systematically excavate the influencing factors and deeply analyze the Lastly relationship among the influencing factors of college students’ online learning. Finally, we discuss and summarize the results of the mixed methods.

## 2. Qualitative Research Design and the Theoretical Model

### 2.1. Grounded Theory and Interview

**Figure 1:**
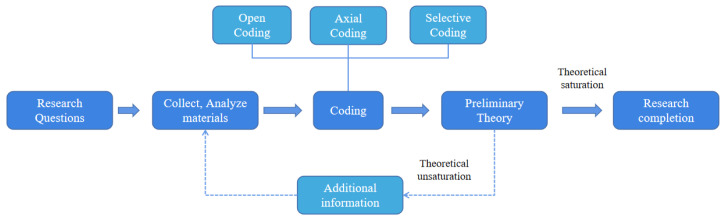
Research steps in GT.

GT is a qualitative research method jointly proposed by Anselm and Barney of Columbia University [17]. As a research methodology, GT can help to better understand the nature and characteristics of problems, and then construct new theories [18]. It is a systematic approach that begins with an inductive query and ends with collecting, analyzing data, and building an underlying theory [19]. During the process of GT, the researcher is required to remain open-minded and conduct a careful evaluation of observations and evidence. Specifically, this process includes three steps: open-ended coding, axial coding, and selective coding. In our study, we use the GT method to identify the influencing factors and the mechanism of online learning for college students. The steps of the GT study are shown in Figure 1.

Nonstructured interviews were used in this study to facilitate the collection of information for the theory building of GT. The interview questions refer to students’ opinions, the obstacles and the effects of online learning, the interaction between teachers and students, suggestions on online learning, etc. A total of 130 college students who experienced multiple rounds of online learning during the COVID-19 pandemic from China were interviewed in this study.

### 2.2. Coding Analysis

During the coding analysis of GT, the qualitative data collected from the interviews were analyzed through open-ended coding, and then through axial coding. The coding process is described below. 

First, we performed open coding with the original online learning interview data in three steps: (1) we identified every word, sentence, and paragraph of the interview data, and then labeled them according to the semantics; (2) the same conceptual category from these labels was classified and named by comparing the similarities and differences between them; (3) the concepts were merged and named according to their similarity to form new categories. In open coding, the labels were in the format of “a + serial number”, the concept code was in the form of “A + serial number”, and the category code was in the form of “AA + serial number”. After merging duplicated labels, we obtained 5147 labels. After merging the duplicated categories, we obtained 41 concepts and 13 corresponding categories (see Table 1).

Second, we performed axial coding by comparing and analyzing the relationships among categories that belong to different levels or types from open coding [20]. In other words, the main categories were formed by classifying the subcategories and relating them. Lastly, four broader core categories (learning expectation, learning quality, learning interaction, and learning result) were obtained by reanalyzing and reviewing the 13 subcategories (see Table 1).

### 2.3. The Construction of a Theoretical Model and the Test of Saturation

#### 2.3.1. The Construction of the Theoretical Model

Through the coding analysis of interview data, four core categories were identified: LE, LQ, LI, and LR. LE refers to students’ expectations of online learning. They expect that various changes can appear in line with their expectations in the learning process to meet their learning needs and habits. LQ refers to the study-related tendencies, attitudes, habits, and styles displayed by students, which are the unique psychological characteristics of students in online learning. LI refers to the communication among peers, between students and teachers, and between students and media in online learning. The LR refers to the improvement of the knowledge, skills, and thinking ability of college students through continuous online learning.

On the basis of the relationships among the four core categories, we constructed the model with three paths.

**Figure 2:**
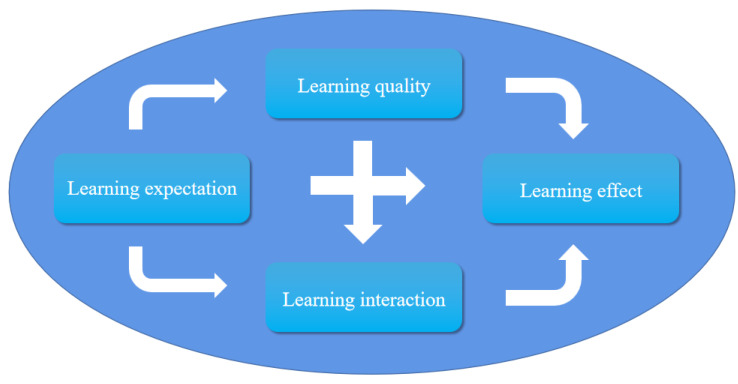
Theoretical model.

Path one: LE→ LQ → LR. As the internal driving force of students’ learning, online LE affects the LQ and the LR. If students’ LE is satisfied, their learning interest may be stimulated, and then they may maintain a good learning attitude, thus ultimately improving their LR. According to respondent no. 48, “I wish teachers were better at using more visually stimulating methods when they teach online, so I would be more interested and it would be easier to remember and understand… If the teacher creates more opportunities for us to interact, then I can participate more seriously and I believe I can gain a lot of skills that I cannot learn from the textbook” (Figure 2).

Path two: LE → LI → LR. If students’ LE is satisfied, the desire for their LI may be stimulated, benign communication with teachers, classmates, and course resources may be easier to maintain, their sense of participation and experience in the course may increase, and the development of the LR may be promoted. According to respondent no. 63, “I hope teachers can provide multiple ways of communication not just limited to the question and answer format during online learning. I like that several teachers use voting, time-limited questions, and anonymous popups to communicate with us so that we can increase our class participation rate and remember our knowledge in a more relaxed and flexible setting” (Figure 2).

Path three: LE → LQ → LI → LR. When the LQ of students is affected by their LE, it may affect the frequency of their class communication and the enthusiasm to participate in the interaction; then, the LR may also have corresponding changes. According to respondent no. 21, “I like teachers who share some extracurricular knowledge and interesting social phenomena with us. I pay more attention and can keep listening carefully to these for a long time. I may participate more actively in the interaction and learn more knowledge and skills in the process of interaction” (Figure 2).

The model diagram constructed in this study can more intuitively, effectively, and comprehensively explain the core categories (influencing factors) and their relationship (operation mechanism) with online learning’s effect on college students (Figure 2).

#### 2.3.2. The Testing of Theory Saturation

To test the theory saturation of our findings, the remaining 19 random interview samples in the basic data were retested. No new concept, logical context, or relationship was generated through the coding and analysis process. The research indicates that the original GT theory fully accommodated relevant concepts and categories, as well as conformed to the theoretical model. Therefore, we considered the theoretical framework to be saturated.

## 3. Quantitative Research Design and Data Analysis

### 3.1. Research Hypotheses

On the basis of the model above, we hypothesized that three core categories would influence students’ online LR: LE, LI, and LQ. On the basis of the three paths we discussed in the model above and the analysis of the interview data, we found that LQ and LI may be related to LE. Therefore, we propose the following hypothesis:

**Hypothesis** **1 (H1).** *The LE affects the LQ.*

**Hypothesis** **2 (H2).** *The LE affects the LI.*

**Hypothesis** **3 (H3).** *The LE affects LR.*

From the interviews, we found that students’ LQ may be related to their LI and LR. Hence, we additionally hypothesize the following:

**Hypothesis** **4 (H4).** *The LQ affects the LI.*

**Hypothesis** **5 (H5).** *The LQ affects the LR.*

The correlation research claimed that learning interactions are essential for constructing new knowledge [21]. On the basis of this research, we hypothesize the following:

**Hypothesis** **6 (H6).** *The LI affects the LR.*

### 3.2. Questionnaire Design and Data Collection

#### 3.2.1. Questionnaire Design

The questionnaire consisted of basic demographic information and variable measures. The demographic information included geographical area, grade level, and ethnicity. The variables measured included LE, LI, LQ, and LR.

#### 3.2.2. Data Collection

We published questionnaires through online survey websites and social media, such as the “Questionnaire Star” survey website, QQ, and WeChat link. A total of 334 valid questionnaires were obtained. The questionnaire’s response rate was over 98%. According to the Chinese government’s administrative geographic system, the percentage of college students in different geographical areas is shown in Table 2: city (33.14%), country (17.44%), township (18.31%), and rural (31.10%). A total of 38 participants (11.05%) were freshmen, while 32 participants (9.30%) were sophomores, 65 (18.90%) were juniors, 177 (51.45%) were seniors, 31 (9.01%) were master’s students, and one (0.29%) was a PhD student. In this survey, ethnic groups included 208 Han (64.07%), 68 Uighur (19.77%), 24 Kazakh (6.98%), seven Mongolian (2.03%), and 37 other ethnic groups (10.76%).

### 3.3. Data Analysis and Results

We evaluated the adequacy of the samples using the SPSS22.0 software. We obtained a standardized reliability coefficient value of 0.958, which was more than 0.9, and the KMO (Kaiser–Meyer–Olkin) value was 0.953, which was more than 0.8 [22]. Bartlett’s sphericity test reached a significance level (*p* < 0.0001) [23], indicating that the sample size was adequate. Next, we tested the reliability and validity using the AMOS 22.0 software to conduct structural equation modeling and hypothesis testing.

#### 3.3.1. Reliability and Validity

Following Kaiser and Hair’s suggestion [24,25], the items with Cronbach’s alpha below the cutoff level of 0.7 or with loadings under 0.5 need to be removed before checking the reliability and validity of the structure. We did not remove any items because all the items reached the standard when we check. Next, we tested Cronbach’s alpha and composite reliability (CR) for structural reliability. The assessment results are shown in Table 3 and Table 4. As shown in Table 3, all constructs had Cronbach’s alpha and composite reliability of 0.844 to 0.925 and 0.863 to 0.926, respectively, higher than the recommended level of 0.7 [25]. 

Next, we tested the convergent and discriminant validity to confirm the construct validity. Table 3 shows that the loading of all constructs exceeded 0.593, and the average variance extracted (AVE) value was higher than 0.558. Therefore, the convergent validity was acceptable. Discriminant validity is the degree of structural uniqueness [26]. To assess the discriminant validity, we calculated and compared the square root of the AVE with its inter-construct correlations (Table 4). The calculations met the accepted criteria, suggesting that the square root of the AVE for each construct was better than its correlation with any other construct. Therefore, the discriminant validity was supported. 

#### 3.3.2. Model Fitting and Hypothesis Testing

Before testing the research model, we checked the degree of fit between the model and the actual data. From Table 5, it can be seen that the fitness index of the whole model met the adaptation criterion, indicating that the degree of fitness was good.

The results in Table 6 show that all hypotheses were verified. LE (β = 0.093, *p*-value < 0.001), LQ (β = 0.515, *p*-value < 0.001), and LI (β = 0.340, *p*-value < 0.001) had a direct influence on LR. LE (β = 0.433, *p*-value < 0.001) and LQ (β = 0.441, *p*-value < 0.01) had an influence on LI. In addition, LE (β = 0.508, *p*-value < 0.001) was a significant predictor of LQ. We can see that all the proposed hypotheses were supported.

**Figure 3:**
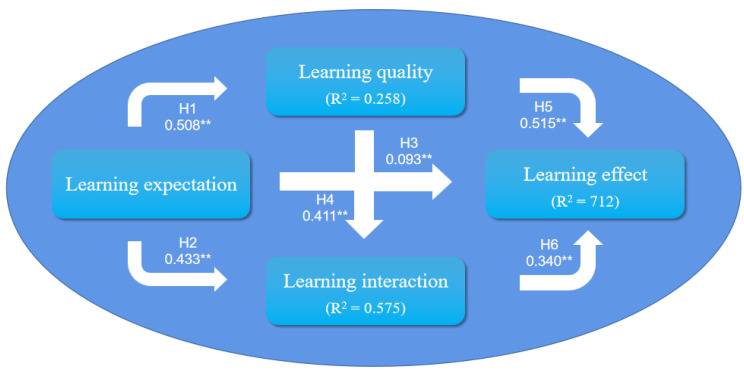
Structural model and R^2^ values.( significant at ** *p* < 0.01).

The endogenous variables of the whole model conformed to an ideal degree: LQ (R^2^ = 0.258), LI (R^2^ = 0.575), and LR (R^2^ = 0.712). It should be noted that 71.3% of the variance in LR was interrupted by the model (Figure 3). The model, therefore, seemed effective for verifying the learning result of college students on online learning. From the path coefficients, the influence of LE (0.508 × 0.515 + 0.508 × 0.441 × 0.340 + 0.433 × 0.340 + 0.093 = 0.578), LQ (0.441 × 0.340 + 0.515 = 0.665), and LI (0.340) on the LR of college students was calculated. It can be seen that LQ had the strongest influence on the LR of college students during online learning.

## 4. Discussion

This study aimed to examine the factors that influence online learning among college students and the relationships among these factors. Some studies attempted to explore the influencing factors of online learning. However, most of these studies were simple and scattered, and few scholars have systematically explored the influencing factors and the internal mechanism relationship between them. We filled this gap by solving two problems. First, we explored the influencing factors of college students’ online learning comprehensively and systematically (research question 1). Second, we verified the mechanism relationship between these influencing factors (research question 2). Results of this study suggest that LE, LQ, LI, and LR are four influence factors of online learning for college students. The results also suggest that LE, LQ, and LI directly influence LR. In addition, LE was the starting factor of the online learning process, whereas LQ and LI were the mediating factors of the online learning process. We discuss three paths to explain their operation mechanism.

### 4.1. The Mediating Role of LQ

As we can see from the result, LQ is a mediating factor between LE and LR during the online learning process. In other words, LE affects the LQ, which in turn affects the LR. Specifically, the college students’ interests and motivation for online learning were easily increased when their LE was appropriately satisfied. Subsequently, college students would keep a good attitude toward their online learning and promote the production of their LR. Our findings confirmed the correlation research [27] that LE was important for building learning goals, motivation, interest in learning, and continuity of learning. They further confirmed the influence between LE and LQ. According to Vroom’s expectancy theory [28] proposed in 1964, the expectation degree and the attractiveness of outcome determine how seriously the student tries to achieve something. The relationship between LE and LQ in our study further supports this theory. In terms of mediating the role of LQ, LQ not only was a relatively stable tendency exhibited by students in the learning process but also played an important role in motivation monitoring, which stimulates learning awareness and regulates it during the learning process [29,30]. The self-regulated learning theory proposed by Zimmerman [31] in 1989 also showed us a significant link between the persistence of student learning and their LR. One student indicated that the secret of their success in study derived from constant interest and motivation, confirming the influence of LQ on the LR. To improve the quality of LR, schools should help students clarify their LE and goals, as well as encourage and guide them to enhance their LQ [32].

### 4.2. The Mediating Role of LI

It is found that LI was a mediating factor between LE and LR during the online learning process. In other words, LE affects the LI, which in turn affects the LR. Specifically, the college student’s desire and demand for interaction with online learning were easily motivated when their LE was appropriately satisfied. Subsequently, college students may be involved in a positive communication environment with teachers, classmates, and course resources. The college student may obtain more knowledge and skill through teacher–student, student–student, and student–media interactions during online learning. As several researchers have pointed out [33], LE was a progressively changing psychological status according to the experience of the students’ constant reaction to information from different sources. Furthermore, students promote their behaviors by perceiving the results of their behaviors. According to Tolman’s symbol learning theory [34], learning is an activity with practical meaning, which is guided by a specific goal and involves the formation of expectations. In other words, learners’ behavior is guided by their expectations, i.e., their behavior is purposeful. In other words, the participation rate and quality of online learning of the student will be affected by their LE. When students’ LE is supported [35], they may show more satisfaction and increase the frequency of LI during online learning. Moreover, teacher–student interaction was one of the most direct positive factors influencing students’ online experience [36]. At the same time, immediate feedback from the teacher was vital to students’ online course performance [37]. Our research findings are consistent with the constructivist learning theory [38], which shows that learning activities are a process for students to achieve meaningful construction of knowledge through collaboration and conversation between teachers and classmates in certain scenarios. According to respondent No. 79, “my teacher always responded to me promptly, and I was so proud to be praised by her for being a brave person to ask questions. I like to introspect and try to solve some difficult things when I am encouraged by my teacher so that I can gain more knowledge and skill beyond the book”. According to the interview, timely feedback to students given by teachers would increase the opportunities for their thinking, communication, and acquisition of LR.

### 4.3. Chain Mediating Role of LQ and LI

We found that LE affects LR through LQ and LI. In other words, the satisfaction of students’ LE would help them to maintain a positive LQ, increase the frequency of LI, and improve the quality of LR during online learning. This conclusion is in accordance with the research results of Huang and Huo [39], which suggested that students form better learning attitudes and habits when their expectations are met, which also helps to increase the positive and frequent interactive engagement and the quality of LR. A study of graduate students [40] also found a chain mediating role of LQ and LI, which mentioned that the change in LE directly affects the change in learning motivation. Furthermore, students will adjust their learning attitudes and behaviors on the basis of their learning motives, which in turn has an impact on LQ. Another study [41] indicated that LE will stimulate students’ learning motivation, increase their LI in different activities such as practice activities, group work, unstructured discussion, and classroom interaction, and then improve their LR. Furthermore, self-regulated learning theory [31] presents a similar view in that students have different achievement goals that affect their choice of learning tasks, persistence in completing learning tasks, the level of effort they exert, and how they perform learning activities. In addition, the chain mediating role of LQ and LI was also confirmed from the interview. According to respondent No. 102, “I hope teachers can share with us more interesting stories, social phenomena, or cross-subject knowledge. I may feel super excited and attracted to this and willing to discuss it with my classmate. I believe we can all obtain more useful information through the class”.

## 5. Implications for Practice

This research explored the influencing factors of online learning and its operation mechanism among Chinese college students during the COVID-19 pandemic, which is conducive to a deep understanding of the process and improving the quality of online learning. According to the theoretical model we constructed and tested, the influencing factors of online learning were LE, LQ, LI, and LR, and LE affected LR through mediating factors LQ and LI. At the same time, we also found the LR was affected by LE through a chain of mediating role factors LQ and LI.

In terms of LE, the improvement in students’ LE can be achieved from the following three aspects: the student’s self-LE, the LE of the teacher, and the environment. Schools should first attach more importance and make every effort to help students to recognize and adjust to their LE [42] so that students can set up reasonable LE. Furthermore, teachers should increase communications with students to improve their understanding of students’ LE [43] and provide timely consultation for students to meet their reasonable learning demands. Moreover, on the basis of the students’ LE of the learning environment, high-quality online teaching platforms and resources should be provided to the student by stakeholders such as families, universities, society, etc. 

From the result of our study, we know that LQ plays an important role in three paths, showing one direct (LQ → LR) and two indirect paths (LE → LQ → LR and LE → LQ → LI → LR). Therefore, it is necessary to pay attention to LQ and explore the strategies to strengthen it during online learning. Four methods may help us to improve LQ in practice. Firstly, teachers were suggested to focus on the individual differences of various students and their needs according to their learning tendency [44], thus providing individualized teaching strategies. Secondly, teachers were proposed to increase the content of student autonomy in learning in their curriculum design so as to stimulate students’ desire and motivation to explore new knowledge [45,46], while students could continuously maintain positive and stable learning attitudes during online learning. Thirdly, students should actively change their passive learning habits [47]. They can also effectively improve and adjust their learning mode with the help of information technology such as multimodal time management assistance technology [48]. Lastly, teachers were also suggested to communicate with the student to thoroughly analyze their learning styles [49] and explore efficient and personalized learning strategies together to improve their LR.

In terms of LI, we found one direct (LI → LR) and one indirect path (LE → LQ → LI → LR) from the result of our study. Some specific suggestions are given to improve the LI through future research. First, teachers and schools need to rethink how to improve the quality of teacher–student interaction, student–student interaction, and media interaction. Ren [46] suggested that teachers should actively engage in emotional communication with the student in various ways and improve their teaching effectiveness by enhancing the depth of course interaction. In order to ensure efficient communication and exchange among students, teachers, and the media, Wang [50] suggested that teachers should positively create interactive opportunities in teaching activities, such as installing exchange and discussion sessions, as well as arranging group cooperation tasks. In addition, teachers should use newly emerging interactive methods. For example, the teacher can use information technology to visualize learning content [51], as well as enhance the hyperlinks of each section of the online course [52], to promote students to build their knowledge system.

## 6. Research Contribution and Limitation

As the relevant research mentioned that the quantitative and qualitative methods could supplement each other and allow for a more comprehensive analysis of the study question [53], we adopted a mixed method in order to realize the complementary role of qualitative and quantitative methods and reach a deeper understanding of the influencing factors of online learning and its operation mechanism among Chinese college students during the COVID-19 pandemic.

The contributions of this study are manifold. Firstly, it collates and deepens the research on the influencing factors of online learning. This paper constructs a theoretical model of the influencing factors of online learning and its operating mechanism, highlighting the following influencing factors: LE, LQ, LI, and LR. Secondly, this study verifies the theoretical model path through the practical data of college students’ online learning. For example, the influencing factors (LE, LQ, and LI) directly affect LR. LQ and LI and their combined relationships mediate the path from LE to LR. Thirdly, it provides some suggestions for the concrete implementation of improving the quality of online learning among Chinese college students during the COVID-19 pandemic according to our research results. Lastly, this study provides a novel hybrid research paradigm for other countries during the COVID-19 pandemic and the globalization of online learning. This paper provides a comparative conclusion reference for the future study of online learning in different countries.

Nevertheless, there were a few limitations to this study. Firstly, some variables and factors were not included in the study due to many reasons, such as time of online learning, Internet penetration, and technology [54]. These variables and factors may be influenced by the respondent’s region epidemic prevention policy and may also lead to the differences in the factors influencing online learning among different countries, which can be further explored in future research. Secondly, GT requires researchers to maintain a sensitive and open attitude in research [55], while the coders of this study may be limited by their existing theoretical experience when they conducted data coding [56]. Thirdly, the sample representativeness should be considered rigorous. The sample of this study was students, whereas the school administrators and teachers were not included. Future research could consider enriching relevant research conclusions from the perspective of other samples;

Lastly, one study [57] found that students’ online learning is likely to be influenced by cultural differences. For example, Korean, Finnish, and American students who differ in their online collaborative behaviors may exhibit different online learning patterns. Although the sample of our study was from China, the findings are similar to those of studies addressing online learning in countries such as Russia, Germany, the United States, and Canada [58,59,60]. For example, in terms of influencing factors, a study by German scholars [59] found positive or negative effects of learning expectations on online learning. In terms of mechanism relationships, a study by Russian scholars [58] found that positive online learning attitudes can help students absorb information. In sum, other countries can further discuss this on the basis of this study combined with their national cultural characteristics.

## 7. Conclusions

With the prevalence of online learning during the COVID-19 pandemic, the learning styles in education around the world changed substantially. This study aimed to construct and test a theoretical model of the influencing factors of online learning and its operation mechanism among Chinese college students through a mixed-method approach. Our research solved the question of factors influencing college students’ online learning and verified the relationships among these factors through qualitative and quantitative methods. The results of the study showed that there are four factors (LE, LQ, LI, and LR) that influence online learning, and there are three factors (LE, LQ, and LI) that have a direct influence on LR. Furthermore, from the perspective of mediating factors, there are three paths to reflect the operation mechanism from LE to LR (LE → LQ → LR, LE → LI → LR, and LE → LQ → LI → LR). In other words, LE was the starting factor of the online learning process, whereas LQ and LI were the mediating factors of the online learning process. Moreover, the generated theoretical model effectively explained the internal operating mechanism that influences college students’ online learning. The model enriches the theory and guides the practice of online learning. Our findings help to improve the quality and effectiveness of online learning for students. This study is noteworthy because its results provide educators and researchers in China and other countries with a new perspective that has implications for higher-education practice.

## Figures and Tables

**Table 1:** Results of coding analysis.

Core Category	Category	Concepts
Learning expectation (LE)	Self-expectation	Anticipation of your learning and results
	Expectation of teacher	Anticipation of the teaching style, content, and evaluation of the lecturer
	The expectation of the environment	Anticipation of your physical environment, mobile devices, online learning platforms, and network quality
Learning quality (LQ)	Learning tendency	Preferences for learning mood, method, content, approach, and environment
	Learning attitude	A state in which behavioral dispositions and psychological responses to learning can be maintained in a more sustained and stable manner
	Learning habit	Automated personal learning behaviors formed and developed through repeated practice
	Learning style	Learning style that is preferred or unique and personal
Learning interaction (LI)	Teacher–student interaction	Interaction and communication between teachers and students for the purpose of promoting student development
	Student–student interaction	Exchange and communication between students to work together on a task or have an intellectual discussion
	Media interaction	Interaction of students with the system of knowledge, courses, and resources
Learning result (LR)	Knowledge	Mental progress through continuous learning and experience
	Skill	Improvement in operational skills acquired through continuous learning and experience
	Thinking ability	Progress in the ability to identify, integrate, understand and apply through continuous learning and experience

**Table 2:** Summary of demographic characteristics of the study sample.

Name	Option	Frequency	Percentage (%)	Accumulative Perception (%)
Geographical area	City	114	33.14	33.14
	Country	60	17.44	50.58
	Township	63	18.31	68.90
	Rural	107	31.10	100.00
Grade	Freshmen	38	11.05	11.05
	Sophomore	32	9.30	20.35
	Junior	65	18.90	39.24
	Senior	177	51.45	90.70
	Master’s student	31	9.01	99.71
	PhD student	1	0.29	100.00
Nationality	Han	208	60.47	60.47
	Uighur	68	19.77	80.23
	Kazakh	24	6.98	87.21
	Mongolian	7	2.03	89.24
	Others	37	10.76	100.00
Total		344	100.0	100.0

**Table 3:** Cronbach’s alpha, loadings, composite reliability, and AVE values.

Construct (No. of Items)	Cronbach’s Alpha	Loadings	CR	AVE
LE(6)	0.923	0.668, 0.789, 0.822, 0.799, 0.821, 0.817	0.924	0.671
LQ(5)	0.921	0.782, 0.703, 0.800, 0.814, 0.746	0.922	0.702
LI(4)	0.844	0.693, 0.713, 0.593, 0.649	0.863	0.558
LR(6)	0.925	0.647, 0.646, 0.764, 0.703, 0.630, 0.624	0.926	0.678

**Table 4:** Assessment of discriminant validity in the case of the analyzed model.

	LQ	LE	LR	LI
LQ	0.838			
LE	0.508	0.819		
LR	0.788	0.579	0.823	
LI	0.661	0.657	0.742	0.760

Note: Diagonal elements are the square root of the AVE.

**Table 5:** The goodness of fit indices for tested models.

Common Indicators	χ²	df	*p*	CMIN/DF	GFI	RMSEA	RMR
Judgment criteria	-	-	>0.05	<3	>0.9	<0.10	<0.05
Value	515.246	183	0.000	2.816	0.871	0.073	0.040
Other indicators	CFI	NFI	NNFI	TLI	IFI	SRMR	RMSEA 90% CI
Judgment criteria	>0.9	>0.9	>0.9	>0.9	>0.9	<0.1	-
Value	0.943	0.915	0.935	0.935	0.943	0.048	0.065~0.080

Default Model: χ^2^ (210) = 6054.579, *p* = 1.000.

**Table 6:** Hypothesis testing of the research model (significant at *** *p* < 0.001, * *p* < 0.05).

Hypothesses	Path Coefficients	Finding
H1: LE⇒LQ	0.508 ***	Supported
H2: LE⇒LI	0.433 ***	Supported
H3: LE⇒LR	0.093 *	Supported
H4: LQ⇒LI	0.441 ***	Supported
H5: LQ⇒LR	0.515 ***	Supported
H6: LI⇒LR	0.340 ***	Supported

P.s.: LLCI is the lower level of the 95% confidence interval; ULCI is the upper level of the 95% confidence interval.

## Data Availability

The data presented in this study are available on request from the corresponding author. The data are not publicly available due to ethical requirements.

## References

[1] Education: From Disruption to Recovery. http//en.unesco.org/covid19/educationreponse.

[2] Cranfield D.J., Tick A., Venter I.M., Blignaut R.J., Renaud K. (2021). Higher Education Students’ Perceptions of Online Learning during COVID-19—A Comparative Study. Educ. Sci..

[3] Salto D. (2020). COVID-19 and higher education in Latin America: Challenges and possibilities in the transition to online education. eLearn.

[4] Andi C., Yusriadi Y., Asma G. (2022). Transformation of the Education Sector during the COVID-19 Pandemic in Indonesia. Educ. Res. Int..

[5] UNESCO The New Normal—What Needs to Be Different than before?. https://en.unesco.org/futuresofeducation/debates/the-new-normal.

[6] Hiltz S.R. (1986). The “virtual classroom”: Using computer-mediated communication for university teaching. J. Commmun..

[7] Singh V., Thurman A. (2019). How Many Ways Can We Define Online Learning? A Systematic Literature Review of Definitions of Online Learning (1988–2018). Am. J. Distance Educ..

[8] Gui X.F. (2022). Meta-Analysis of the Effect of Online Learning and the Influence Mechanism of Junior High School Students’ Online Learning during COVID-19 Pandemic—From the Perspective of Techenology Acceptance Model.

[9] Jing Y.J., Li X., Jiang X. (2021). Analysis of Factors that Influence Behavioral Intention to Use Online Learning and Enlightenment in the Post COVID-19 Education. China Educ. Technol..

[10] Alismaiel O.A., Cifuentes-Faura J., Al-Rahmi W.M. (2022). Online Learning, Mobile Learning, and Social Media Technologies: An Empirical Study on Constructivism Theory during the COVID-19 Pandemic. Sustainability.

[11] Boca G.D. (2021). Factors Influencing Students’ Behavior and Attitude towards Online Education during COVID-19. Sustainability.

[12] Agormedah E.K., Henaku E.A., Ayite D.M., Ansah E.A. (2020). Online Learning in Higher Education during COVID-19 Pandemic: A case of Ghana. J. Educ. Technol. Online Learn..

[13] Sarfraz M., Hussain G., Shahid M., Riaz A., Muavia M., Fahed Y.S., Azam F., Abdullah M.T. (2022). Medical Students’ Online Learning Perceptions, Online Learning Readiness, and Learning Outcomes during COVID-19: The Moderating Role of Teacher’s Readiness to Teach Online. Int. J. Environ. Res. Public Health.

[14] Miao J., Ma L. (2022). Students’ online interaction, self-regulation, and learning engagement in higher education: The importance of social presence to online learning. Front. Psychol..

[15] Singh S., Roy D., Sinha K., Parveen C., Sharma G., Joshi G. (2020). Impact of COVID-19 and lockdown on mental health of children and adolescents: A narrative review with recommendations. Psychiatry Res..

[16] Wang X., Zhang L., Yang W.Y., Lu X., Xu W.W., Gao Z. (2021). How Online Learning Resources Affect Academic Emotions and Learning Outcomes—A Meta-Analysis Based on the Control-Value Theory. Mod. Dist. Educ. Res..

[17] Glaser B.G., Strauss A.L., Strutzel E. (1968). The discovery of grounded theory; strategies for qualitative research. Nurs. Res..

[18] Anne S.T., Zhang Z.X. (2011). Management and Building Theory: The Strategies of Research on Chinese Native Management. Chongqing Daxue Xuebao.

[19] Charmaz K. (2012). The power and potential of grounded theory. Med. Soc. Online.

[20] Kurucay M., Inan F.A. (2017). Examining the effects of learner-learner interactions on satisfaction and learning in an online undergraduate course. Comput. Educ..

[21] Linstone H.A., Turoff M. (1975). The Delphi Method: Techniques and Applications.

[22] Pallant J. (2013). SPSS Survival Manual.

[23] Chen D., Xiang P., Jia F., Zhang J., Liu Z. (2020). An indicator system for evaluating operation and maintenance management of mega infrastructure projects in China. Int. J. Environ. Res. Public Health.

[24] Kaiser H.F. (1974). An index of factorial simplicity. Psychometrika.

[25] Hair J.F., Black W.C., Babin B.J., Anderson R.E. (2010). Multivariate Data Analysis: A Global Perspective.

[26] Fan B. (2013). Achieving horizontal integration of municipal e-government in China: Assessment of managerial mechanisms. Inf. Dev..

[27] Wu Z.M., Li Y. (2019). A Policy Study on Students’ Learning Expectations Viewed from Respect: A Case of Shanghai. J. East China Norm. Univ..

[28] Vroom V.H. (1994). Work and Motivation.

[29] Davis F.D. (1989). Perceived usefulness, perceived ease of use, and user acceptance of information technology. MIS Q..

[30] Wang Y. (2021). School Readiness Approaches to Learning and Classroom Engagement: A Longitudinal Study. J. Shanghai Educ. Res..

[31] Zimmerman B.J. (1989). A social cognitive view of self-regulated academic learning. J. Educ. Psychol..

[32] Zhang P.G., Qian Z., Jiang L.D. (2019). Comparative Study on College Students’ Learning Expectation and Learning Habits Based on Theory of Zimmerman’s Self-regulated Learning. Advances in Social Science, Education and Humanities Research, VOL.310, Proceedings of the 3rd International Conference on Culture, Education and Economic Development of Modern Society (ICCESE 2019), Moscow, Russia, 1–3 March 2019.

[33] Liang N.J., Yin F., Wu M.Z. (1999). A Research on the pelationshipe between students’ self -expected and teacher-encouraged mentalraits. Psychol. Sci..

[34] Tolman E.C. (1948). Cognitive maps in rats and men. Psychol. Rev..

[35] Yuen H.K., Fox R., Sun A., Deng L. (2009). Course management systems in higher education: Understanding student experiences. J. Edu..

[36] Jiang Y.J., Bai X.M., Wu W.C., Luo X.J. (2019). Research on the Structural Relationship of Influencing Factors of Online Learning Experience. Mod. Distance Educ. Res..

[37] Anderson T. (2003). Getting the mix right again: An updated and theoretical rationale for interaction. IRRODL.

[38] Tennyson R.D., Volk A. (2015). Learning theories and educational paradigms. International Encyclopedia of the Social & Behavioral Sciences.

[39] Huang S., Huo L.Y. (2014). The Main Factors Affecting Children’s Approaches to Learning: Research Progress and Implications. Comp. Educ. Rev..

[40] Li Z. (2002). On the Cognitive and Behavioral Choices of Current Graduate Students. Acad. Deg. Gr. Educ..

[41] He G.W. (2012). An analysis of the development and stimulation of learning motivation. Educ. Voca..

[42] Tang J. (2014). The Research on the Learning Expectations and Learning Outcomes of College Students.

[43] Yao S.X. (2015). Influence of learning expectations on assessment of teaching and its response. Educ. Rev..

[44] Yang M.P., Jin X. (2020). Learning quality: The intrinsic need for college students to improve the quality of learning. Heilongjiang Educ..

[45] Ma L.P., Cao Y.L. (2020). Classroom Interaction and Course Satisfaction in Synchronous Online Education—Taking the Ed. D in Graduate School of Education of Peking University for Example. Mod. Educ. Technol..

[46] Ren Y. (2021). Research on the Influencing Factors of Online Learners’ Continuous Learning Intention:Based on the ECM Perspective. J. Open Learn..

[47] Zhang Y.Q. (2018). Investigation Report on the Current Situation of Independent Learning of College Students in the Less Developed Western Region under the Internet Environment: Five Universities in Gansu Province as an Example. China Newsp. Ind..

[48] Zhu C.L. (2021). Research on Learning Experiences in Online Open Education Courses. Adl. Educ..

[49] Parra B.J. (2016). Learning strategies and styles as a basis for building personal learning environments. Int. J. Educ. Technol. High. Educ..

[50] Wang S.Y. (2021). An Exploration of the Relationships between Interactive Forms of Online Course Teaching and Students’ Learning Engagement. J. East China Norm. Univ..

[51] Jiang Q., Zhao W., Wang P.J., Wang L.P. (2015). Realization of Individual Adaptive Online Learning Analysis Model Based Big Data. China Educ. Technol..

[52] Wei S.P. (2012). An Analysis of Online Learning Behaviors and Its Influencing Factors: A Case Study of Students’ Learning Process in Online Course “Open Education Learning Guide” in the Open University of China. Open Educ. Res..

[53] Greene J.C., Caracelli V.J., Graham W.F. (1989). Toward a conceptual framework for mixed-method evaluation designs. Educ. Eval. Policy Anal..

[54] Clothey R. (2010). Current trends in higher education: Expanding access in Asia Pacific through technology. J. Comp. Int. High. Educ..

[55] Johnson B., Turner L.A., Tashakkori A., Teddie C. (2003). Data collection strategies in mixed method research. Handbook of Mixed Methods in Social and Behavioral Research.

[56] Johnson R.B., Onwuegbuzie A.J. (2004). Mixed Methods Research: A Research Paradigm Whose Time Has Come. Educ. Res..

[57] Kim K.-J., Bonk C.J. (2002). Cross-cultural Comparisons of Online Collaboration. J. Comput.-Mediat. Commun..

[58] Dikaya L.A., Avanesian G., Dikiy I.S., Kirik V.A., Egorova V.A. (2021). How Personality Traits Are Related to the Attitudes Toward Forced Remote Learning During COVID-19: Predictive Analysis Using Generalized Additive Modeling. Front. Educ..

[59] Hoss T., Ancina A., Kaspar K. (2021). Forced Remote Learning During the COVID-19 Pandemic in Germany: A Mixed-Methods Study on Students’ Positive and Negative Expectations. Front. Psychol..

[60] Nguyen T., Netto C., Wilkins J.F., Bröker P., Vargas E.E., Sealfon C., Puthipiroj P., Li K.S., Bowler J.E., Hinson H.R. (2021). Insights Into Students’ Experiences and Perceptions of Remote Learning Methods: From the COVID-19 Pandemic to Best Practice for the Future. Front. Educ..

